# Metamorphic evolution of amphibolite from Proto-Tethys South Altyn orogen and its geological significance

**DOI:** 10.1038/s41598-026-44259-0

**Published:** 2026-03-17

**Authors:** Shihao Zhang, Tuo Ma, Yongsheng Gai, Liang Liu

**Affiliations:** State Key Laboratory of Continental Evolution and Early Life, Department of Geology, Northwest University, Xi’an, 710069 China

**Keywords:** Amphibolite, *P*–*T*–*t*, LP*–*MP rocks, Continental deep subduction, South Altyn, Planetary science, Solid Earth sciences

## Abstract

Medium- to low-grade metamorphic (LP–MP) rocks, though major constituents of collisional orogens, remain less studied than high-to ultrahigh-pressure (HP*–*UHP) counterparts. Their peak conditions and *P–T–t* evolutions are poorly constrained due to lack of diagnostic assemblages. This study investigates amphibolites enclosed in HP pelitic granulites in Munabulake in South Altyn (SA) HP–UHP belt, unveiling a two-stage metamorphism through inclusion analysis and thermodynamic simulation. The eclogite-facies metamorphism (firstly identified in the westernmost SA) is evidenced by zircon-hosted garnet + omphacite + rutile inclusions and flat heavy rare earth elements (HREEs) patterns without negative Eu anomalies, yielding a peak age of 501.6 ± 2.7 Ma. The amphibolite-facies metamorphism is recorded by titanite-hosted amphibole + plagioclase inclusions, yielding a retrograde age of 437 ± 6.2 Ma and *P*–*T* conditions of 3.7–6.1 kbar/640–725 °C. These findings demonstrate that the amphibolite originated from retrograde metamorphism of eclogite. Integrated with previous studies, our results reveal potentially extensive HP–UHP exposures across the SA, with most rocks attaining eclogite-facies conditions at ~ 500 Ma, documenting an entire continental deep subduction of SA during Early Paleozoic. And the heterogeneous spatial-temporal distribution of metamorphic rocks across different grades in SA reflects differential exhumation processes or variable intensities of retrograde overprinting.

**Supplementary Information:**

The online version contains supplementary material available at 10.1038/s41598-026-44259-0.

## Introduction

Collision orogenic belts, as natural laboratories for investigating material recycling, thermal evolution, and geodynamics of convergent margins, offer key insights into continental crustal subduction and exhumation through HP*–*UHP rocks exposures^1–4^. Globally, HP*–*UHP rocks have been identified in over 30 major orogenic belts, preserving comprehensive records of metamorphic evolution under extreme pressure–temperature (*P–T*) conditions during continental collision^5–7^. Given the global prevalence of HP*–*UHP rocks in orogenic belts, some suggest that continental slabs may undergo unified deep subduction and exhumation, commonly subjected to HP–UHP metamorphism^5,8,9^. However, a key feature of collisional orogens is the close spatial association of LP*–*MP metamorphic rocks (e.g., amphibolite, quartz/mica/chlorite schist) with HP*–*UHP metamorphic rocks. These LP*–*MP rocks lack diagnostic HP index minerals (e.g., coesite, kyanite), obscuring their potential deep subduction history, and hindering understanding of HP*–*UHP terranes’ subduction/exhumation dynamics. Thus, studying LP*–*MP rocks’ metamorphism and their genetic links with associated HP*–*UHP rocks is critical for advancing continental deep subduction models.

Current research classifies closely associated HP*–*UHP and LP*–*MP rocks into three genetic types based on their metamorphic contrasts: (i) Non-subduction-type, LP*–*MP rocks did not experience slab subduction, which may represent tectonic mélanges scraped off^10–12^ and accreted above the subduction zone or overriding plate fragments entrained during exhumation^13,14^; (ii) Shallow subduction-type, LP*–*MP rocks undergo slab subduction but do not reach HP*–*UHP conditions, which may originate from dragged upper-plate materials during shallow subduction^15^ or represent crustal slices detached at varying depths during subduction^3,16^; (iii) Retrograde modification-type, LP–MP rocks, originally subducted to HP–UHP conditions, retain only granulite- to amphibolite-facies assemblages due to complete retrograde overprinting^17–19^, with their HP history preserved solely in micro-scale features^20,21^. In summary, determining whether LP*–*MP rocks closely associated with HP–UHP rocks experienced deep subduction, and classifying their genetic types, provides crucial insights into: (1) Processes and mechanisms of continental deep subduction; (2) Polyphase metamorphic overprinting within subduction channels; and (3) Differential exhumation pathways of HP*–*UHP rocks.

The South Altyn Tagh (SAT), an orogen experienced continental deep to ultra-deep subduction in NW China^21–23^, exhibits eclogite-facies metamorphism in localities like Jianggalesayi^24^, Younusisayi^25^, Danshuiquan^26^, Bashiwake^27^, and Yaganbuyang^28^. However, the prevalent LP*–*MP rocks remain understudied. Particularly in the Munabulake area, only HP granulite-facies metamorphism has been definitively identified^29^. Detailed metamorphic investigations, particularly for potential higher-grade units in Munabulake, are essential to refine the region’s metamorphic framework and elucidate deep subduction-exhumation processes.

This study systematically investigates the LP amphibolites from Munabulake in SA, revealing their metamorphic evolution through detailed field investigations integrated with petrological, mineralogical, thermodynamic modelling, zircon and titanite geochronology with inclusion analyses. We constrain a complete *P–T–t* path demonstrating eclogite-facies metamorphism followed by intense retrogression overprinting. Through comparative analysis with LP–MP rocks across SA, the study synthesizes their genetic characteristics. We propose that the SA continental slab underwent unified subduction, but experienced differential exhumation and retrogression modification, resulting in the distinct spatial-temporal distribution of variably-grade metamorphic rocks.

## Geological background and sample location

**Fig. 1:**
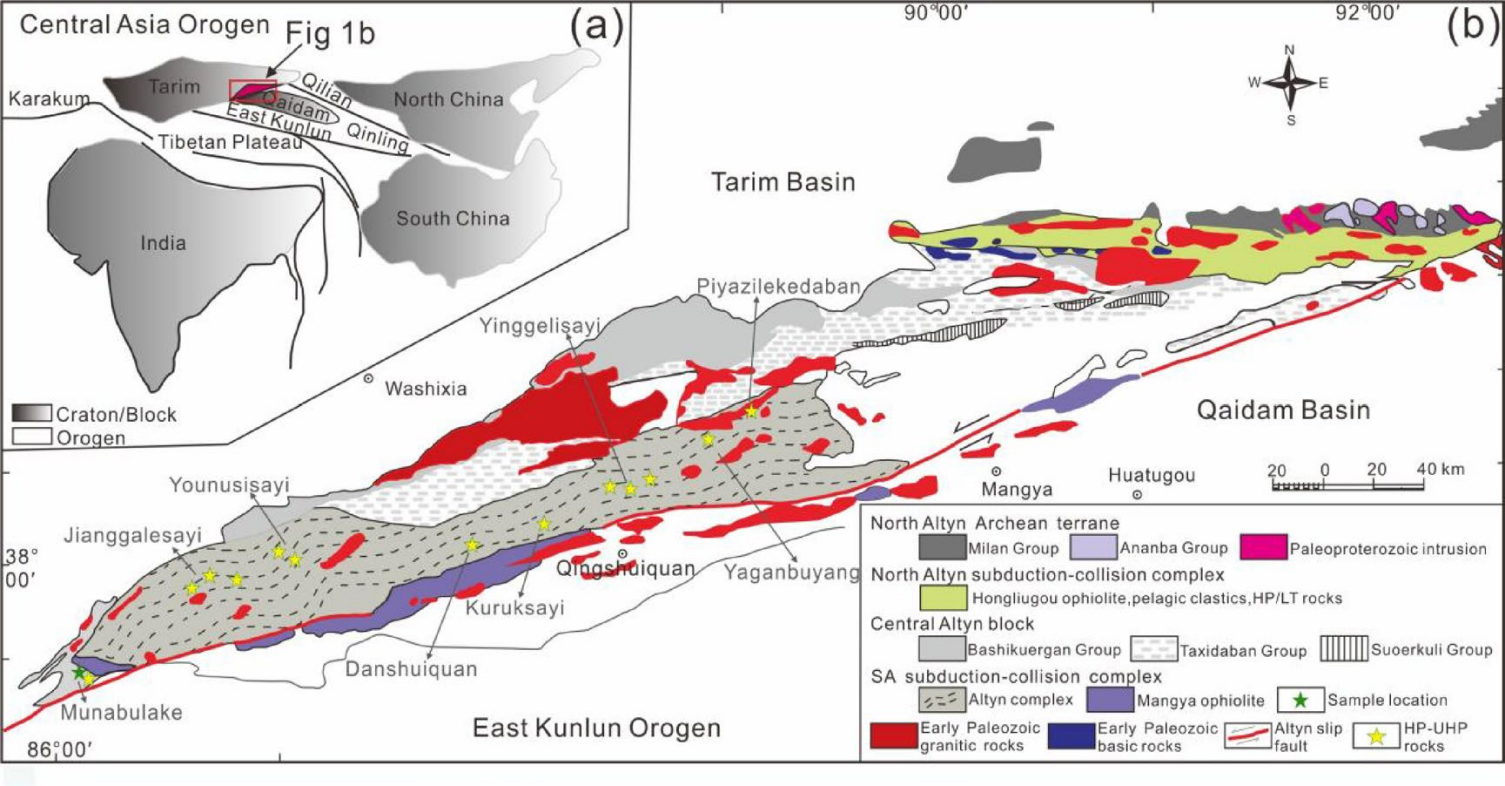
(**a**) Location and tectonic setting map of the Altyn Tagh and adjacent areas (modified by Wang et al.^34^). (**b**) Simplified geological map of the Altyn Tagh, showing the main tectonic units, reported HP–UHP rocks and sample location (modified by Wang et al.^34^).

The Altyn Tagh is situated on the northeastern margin of the Tibetan Plateau. It is bounded by the Tarim block to the north, the Kunlun belt to the south, the Qaidam block to the east, and the Dunhuang orogen to the northeast (Fig. 1a). The Altyn Tagh comprises four tectonic units from north to south: (I) North Altyn Archean terrane; (II) North Altyn subduction–collision complex; (III) Central Altyn block; and (IV) SA subduction–collision complex^30,31^(Fig. 1b).

The North Altyn Archean terrane, regarded as a component of the Tarim Craton basement, comprises the Milan Group (2.9–2.3 Ga felsic gneiss, mafic granulite, amphibolite, marble)^32–34^, unconformably overlying the Annanba Group (Paleoproterozoic unmetamorphosed clastics)^35^, and intruded by 2.3–1.9 Ga Paleoproterozoic plutons^33^.

The North Altyn subduction–collision complex comprises early Paleozoic ophiolites (dated 524–479 Ma with MORB/OIB affinities)^36–38^, deep- to semi-deep-marine clastic rocks, carbonate sequences, volcanic rocks, and LT/HP metamorphic rocks. The HP/LT units, which include blueschists and eclogites formed between 512 and 490 Ma, provide direct evidence for the subduction of North Altyn oceanic lithosphere.

The Central Altyn block, also known as the Milanhe–Jinyanshan block, represents a Meso- to Neoproterozoic sedimentary cover sequence, which primarily composed of arenaceous rocks and marbles (Changchengian Bashikuergan Group) overlain by thick laminated limestone (Jixianian Taxidaban Group)^34^.

The SA subduction–collision complex comprises the SA ophiolitic mélange belt and the SA HP–UHP metamorphic belt. The SA ophiolitic mélange belt is mainly composed of mafic to ultramafic rocks and a suite of flysch sediments. Recent geochronological constraints by Yao et al.^39^ indicate that the ophiolite formed at ~ 518 Ma. The HP–UHP rocks extend across the Altyn Complex, with key exposures from Munabulake^29^, Jianggalesayi^23^, Younusisayi^25^, Danshuiquan^26^, Kuruksayi^40^, Bashiwake^41^, Yaganbuyang^28^, and Piyazikelidaban. Rock types include eclogite (coesite-bearing), retrogressed eclogite, garnet clinopyroxenite, garnet amphibolite, garnet-bearing felsic gneiss, garnet/kyanite metapelite gneiss, magnesite-bearing garnet lherzolite, garnetite, and diverse granulites. Geochronology constrains peak metamorphism to 504–486 Ma, reflecting Early Paleozoic continental deep subduction^31,42,43^.

**Fig. 2:**
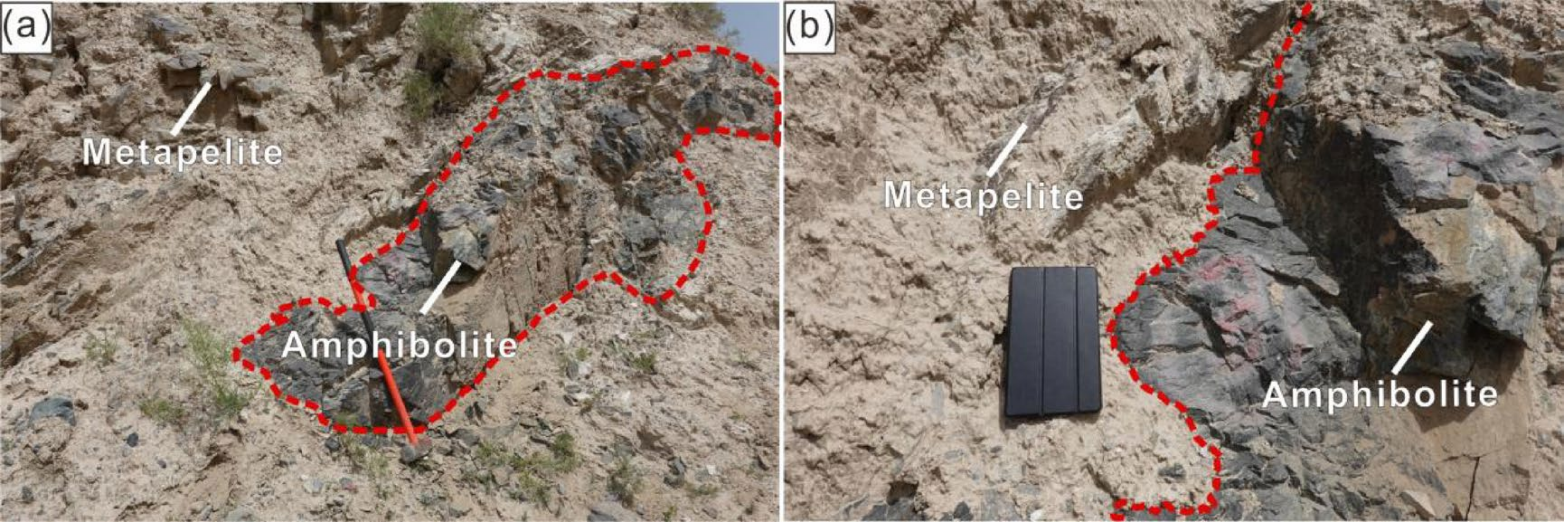
Field photographs showing amphibolite lenses within pelitic gneiss in the Munabulake area.

The study area, Munabulake, is located at the western terminus of the Altyn strike-slip fault within the Altyn–East Kunlun transition zone. It exposes the Bashikuergan Group composed of intrusive and volcanic rocks, and is bordered to the northeast by a mafic–ultramafic mélange. Previously identified HP pelitic granulites (*P* > 11 kbar) show metamorphic ages matching those of the SA HP-UHP belt^29,44^. The studied foliated amphibolites occur as lenses within HP pelitic gneiss (Fig. 2a, b).

## Analytical methods and results

Mineral and bulk-rock compositions, Laser Raman spectroscopy and zircon U–Pb dating were carried out at the State Key Laboratory of Continental Dynamics and Early Life Evolution, Northwest University, China; while titanite U–Pb dating was conducted at Wuhan Sample Solution Analytical Technology Company, China. The analytical methods are described in Supplementary material S1. Mineral abbreviations follow Whitney and Evans^45^.

### Petrography and mineral chemistry

**Fig. 3:**
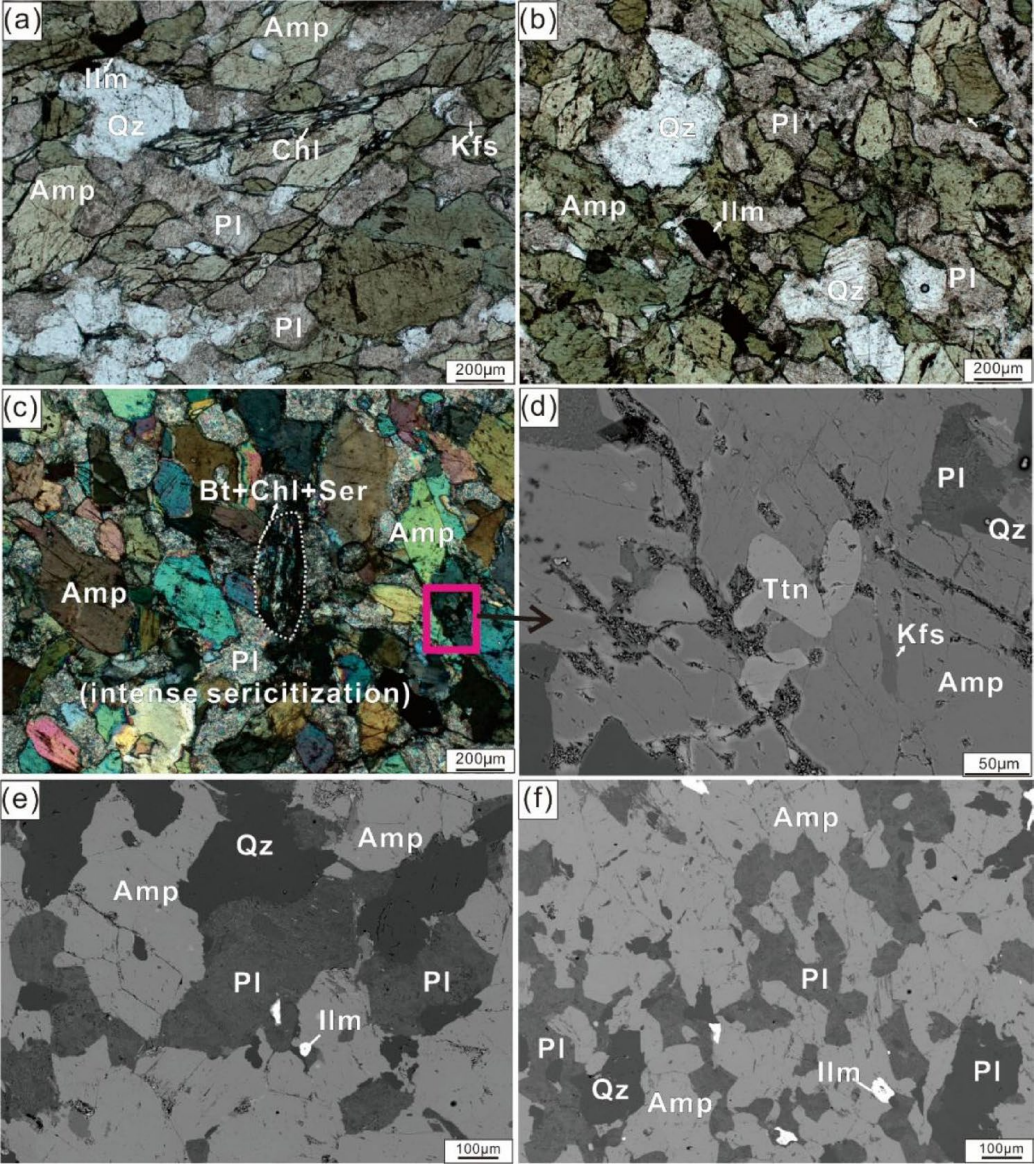
Micrographs of the amphibolite from Munabulake. (**a**,**b**) plane-polarized light (PPL) images; (**c**) Cross-polarized light (XPL) images; (**d**-**f**) back-scattered electron (BSE) images.

The amphibolite exhibits granoblastic structure, and is primarily composed of amphibole (50–55 vol%), plagioclase (30–35 vol%), and quartz (10–15 vol%), with minor K-feldspar, biotite, ilmenite, titanite and chlorite (Fig. 3). Textures and compositions of major mineral phases are detailed below.

**Fig. 4:**
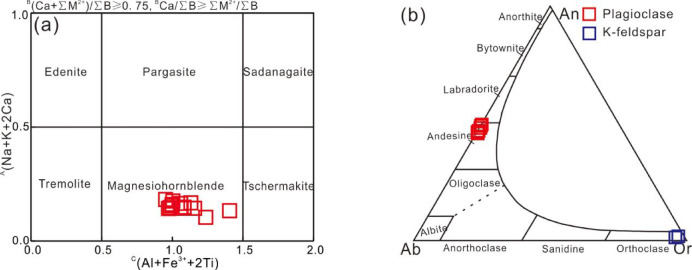
(**a**) Amphibole classification diagram (after Hawthorne et al.^46^); (**b**) Classification diagram for plagioclase: Or = K/ (Ca + Na + K), An = Ca/ (Ca + Na + K), Ab = Na/ (Ca + Na + K).

The amphiboles occur as subhedral to anhedral grains (Fig. 3a–c) and are compositionally similar, with Si = 6.78–6.93 per formula unit (p.f.u.), Mg^#^ = 0.63–0.72, Ti^M2^ = 0.081–0.124 p.f.u., and Al^M2^ = 0.457–0.574 p.f.u. Following the classification of Hawthorne et al.^46^, they are calcic amphiboles and plot entirely within the magnesio-hornblende field (Fig. 4a). Plagioclase occurs as subhedral to anhedral grains (Fig. 3a–c), showing dominant andesine (An_45–49_Ab_51–54_Or_0–1_) (Fig. 4b). K-feldspar is very scarce, occurring as subhedral to anhedral grains that are strongly sericitized, with a composition of An₀Ab₀Or₁₀₀ (Fig. 4b). Biotite is also very rare, appearing as subhedral to euhedral flakes, and is almost completely altered to chlorite and sericite (Fig. 3c). Quartz occurs as anhedral granular grains in equilibrium with amphibole and plagioclase. Major-element compositions of the minerals are listed in Table S1.

### Bulk–rock geochemistry

Major and trace element compositions of the amphibolites are listed in Table S2.

**Fig. 5:**
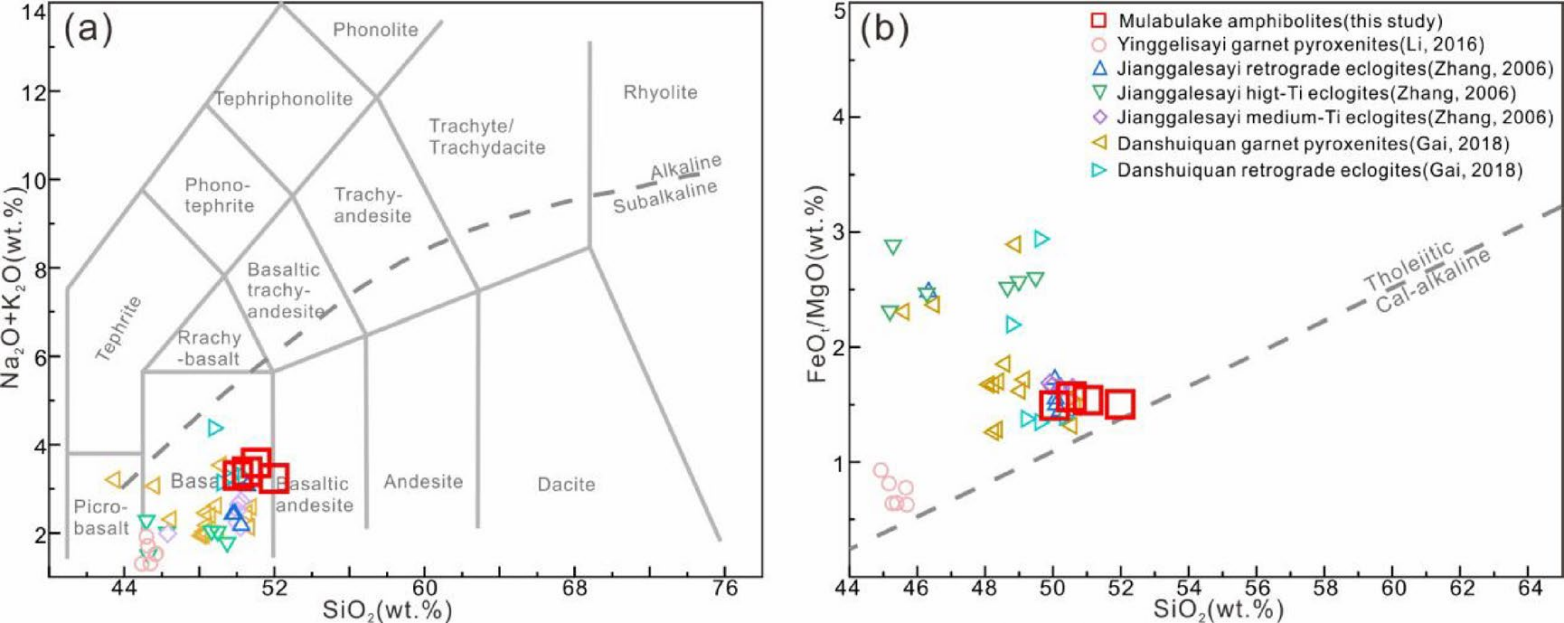
Major element classification diagrams for metamafic rocks from SA. (**a**) TAS diagram; (**b**) FeO^T^/MgO vs. SiO₂ diagram.

The amphibolite exhibits basaltic compositions, with SiO₂ (50.05–51.98 wt%), TiO₂ (1.42–1.52 wt%), Al₂O₃ (13.64–14.15 wt%), FeO^T^ (10.60–11.10 wt%), MgO (7.02–7.10 wt%), and Na₂O (1.42–2.37 wt%), and Mg^#^ of 57–59. These chemical contents consistently classify the rocks as tholeiitic basalts (Fig. 5a, b), as evidenced by their positions in total alkali versus silica (TAS) diagram^47^ and FeO^T^/MgO vs. SiO₂ diagram^48^.

**Fig. 6:**
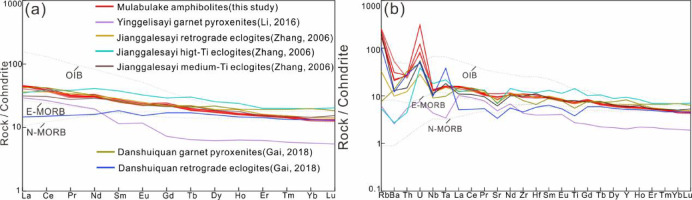
Chondrite-normalized REE patterns and primitive-mantle-normalized trace element patterns for metamafic rocks from SA.

The amphibolites exhibit coherent trace element characteristics marked by: (a) flat chondrite-normalized REE patterns (Σ_REE_ = 70.72–77.48 ppm) with moderate light REEs (LREEs)/HREEs fractionation (La_N_/Yb_N_ = 3.04–3.45; Σ_LREE_/Σ_HREE_ = 2.88–3.02) and negligible Eu anomalies (Eu/Eu* = Eu_N_ /√(Sm_N_ × Gd_N_), 0.92–0.95) (Fig. 6a), resembling OIB/E-MORB signatures; (b) primitive mantle-normalized patterns showing pronounced large ion lithophile elements (LILEs: Rb, Ba, U) enrichment but no significant high field strength elements (HFSEs: Nb, Ta, Ti) depletion (Fig. 6b); and (c) diagnostic trace element ratios (Nb/Zr = 0.07–0.09; Ta/Hf = 0.19–0.23; La/Nb = 1.03–1.15) consistent with within-plate basalt (WPB) affinities.

### Zircon dating

**Fig. 7:**
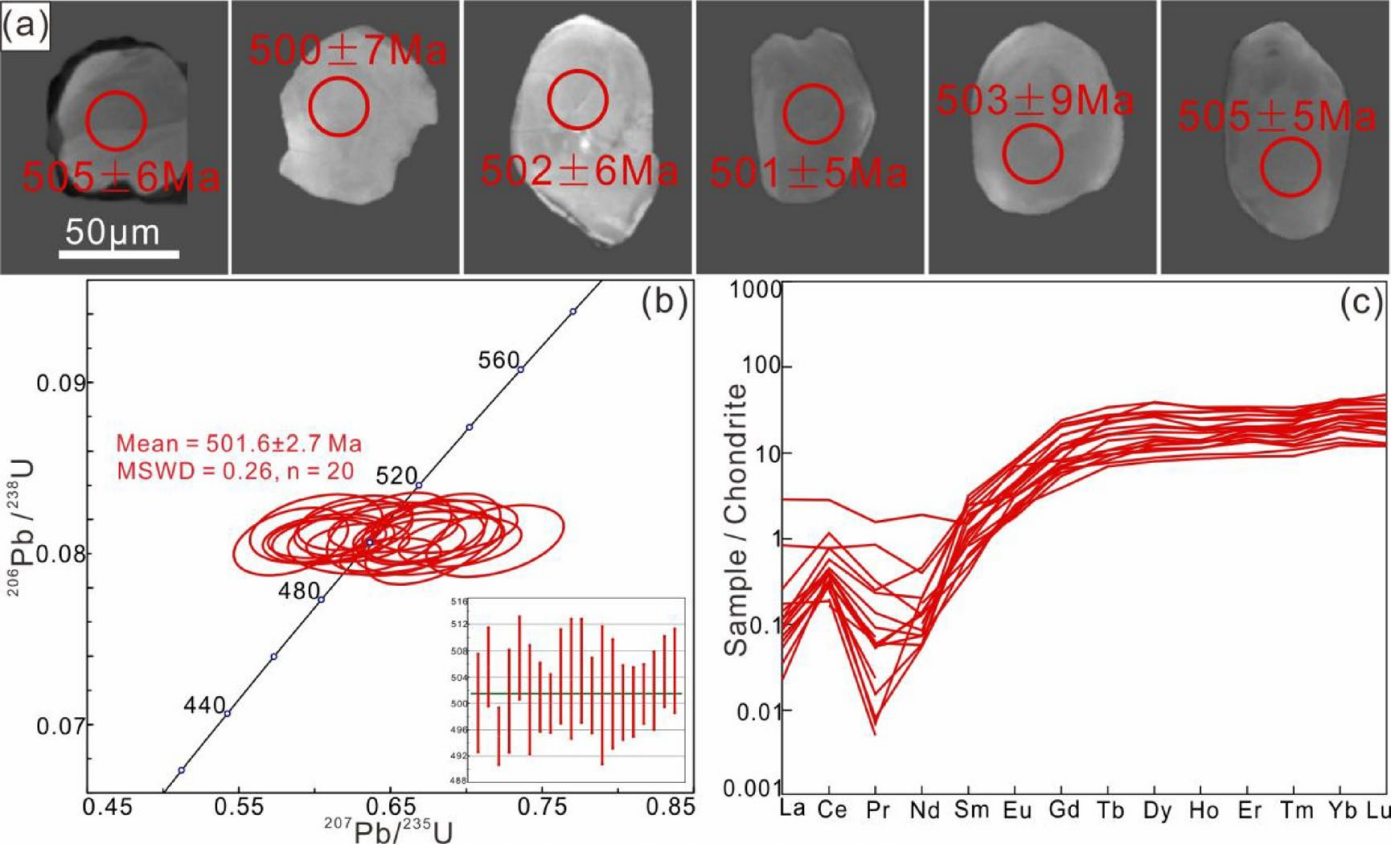
zircons data of amphibolite from Munabulake. (**a**) Cathode luminescence (CL) images of representative zircons; (**b**) U–Pb concordia diagram; (**c**) Chondrite-normalized REE patterns.

Zircons from amphibolite exhibit rounded to granular morphologies with grain sizes of 50–70 μm (Fig. 7a). Cathodoluminescence (CL) imaging reveals diagnostic metamorphic textures including sector zoning, patchy zoning, and weakly zoned to homogeneous domains, consistent with established metamorphic zircon criteria^49–51^. Some grains exhibit core–rim structures, both with homogeneous patchy, nebulous or sector zoning (Fig. 7a), suggesting multi-stage metamorphic growth. However, the extremely narrow rim widths (3–8 μm) preclude reliable U–Pb dating of these metamorphic overgrowths.

A total of 30 zircon spots were analysed, obtaining 29 valid data points (Table S3, S4) and one invalid data point (discarded by the laboratory due to high ^204^Pb background values). Among them, 20 analyses with concordance values greater than 90% were considered reliable and used for age calculation and plotting. The remaining 9 analyses with concordance lower than 90% were excluded due to possible Pb loss or analytical disturbance. The U–Pb analytical spots plot on or close to the concordia line, forming a ²⁰⁶Pb/²³⁸U age groups range from 505 to 495 Ma (Fig. 7b), and yield a weighted mean age of 501.6 ± 2.7 Ma (MSWD = 0.26; Fig. 7b).

Zircon trace-elements data (Table S4) from 20 spots show low REE contents (Σ_REE_ = 9.32–33.37 ppm) and flat HREE pattern, with low Th/U ratios (< 0.03) and unconspicuous Eu anomalies (Fig. 7c).

### Titanite dating

**Fig. 8:**
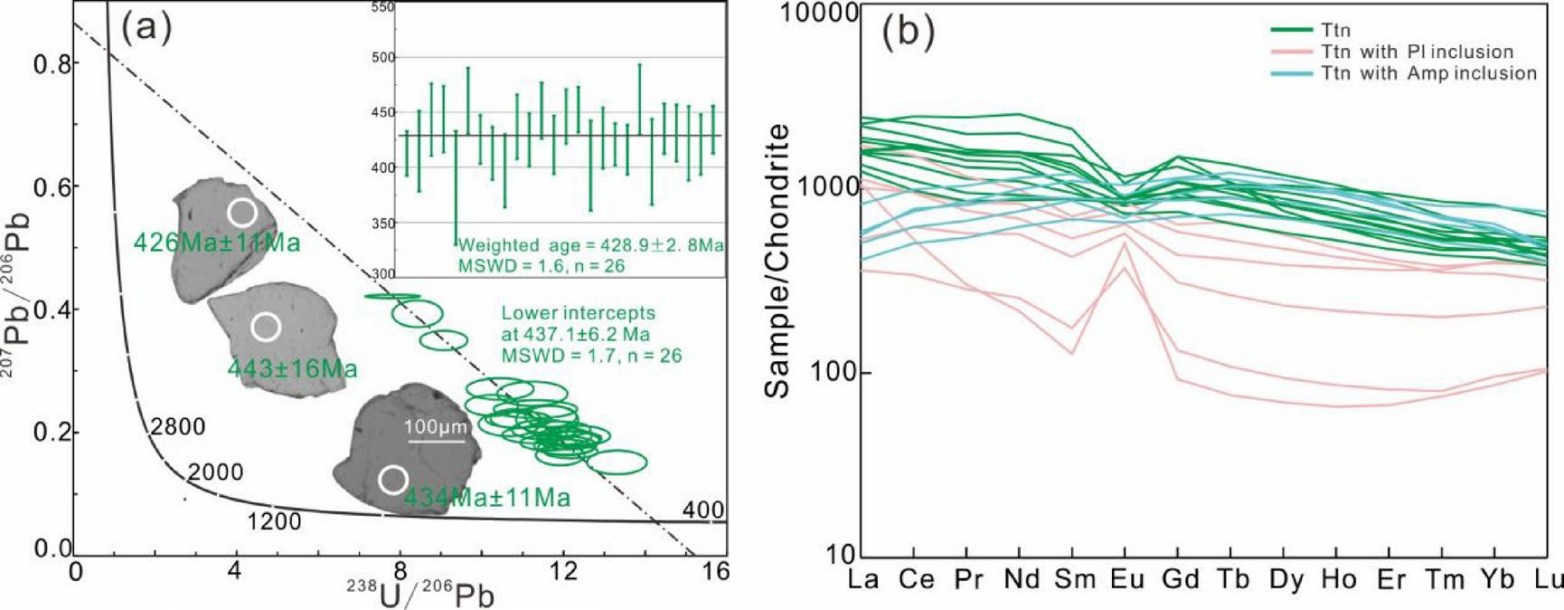
Titanite U–Pb concordia diagrams (**a**) and chondrite-normalized REE patterns (**b**) of amphibolite from Munabulake.

Titanites from amphibolite are subhedral to euhedral crystals (80–300 μm) displaying homogeneous concentric zoning in BSE images (Fig. 8a), indicative of single-stage growth.

A total of 26 titanite spots were analysed (Table S5), yielding a lower intercept age of 437 ± 6.2 Ma (Fig. 8a). After ^207^Pb correction, the ^206^Pb/^238^U weighted age was calculated as 428.9 ± 6.8 Ma (*n* = 26, MSWD = 1.6) (Fig. 8a). These two ages are consistent within analytical uncertainty.

Most titanite grains exhibit a right-declining pattern with slight LREE enrichment and negative Eu anomaly (Fig. 8b; Table S6). Notably, some titanite grains exhibit a convex REE pattern, which may be influenced by amphibole inclusions; and some grains display a right-declining pattern with a positive Eu anomaly, likely due to the presence of plagioclase inclusions (Fig. 8b).

### Inclusion characteristics of zircon and titanite

**Fig. 9:**
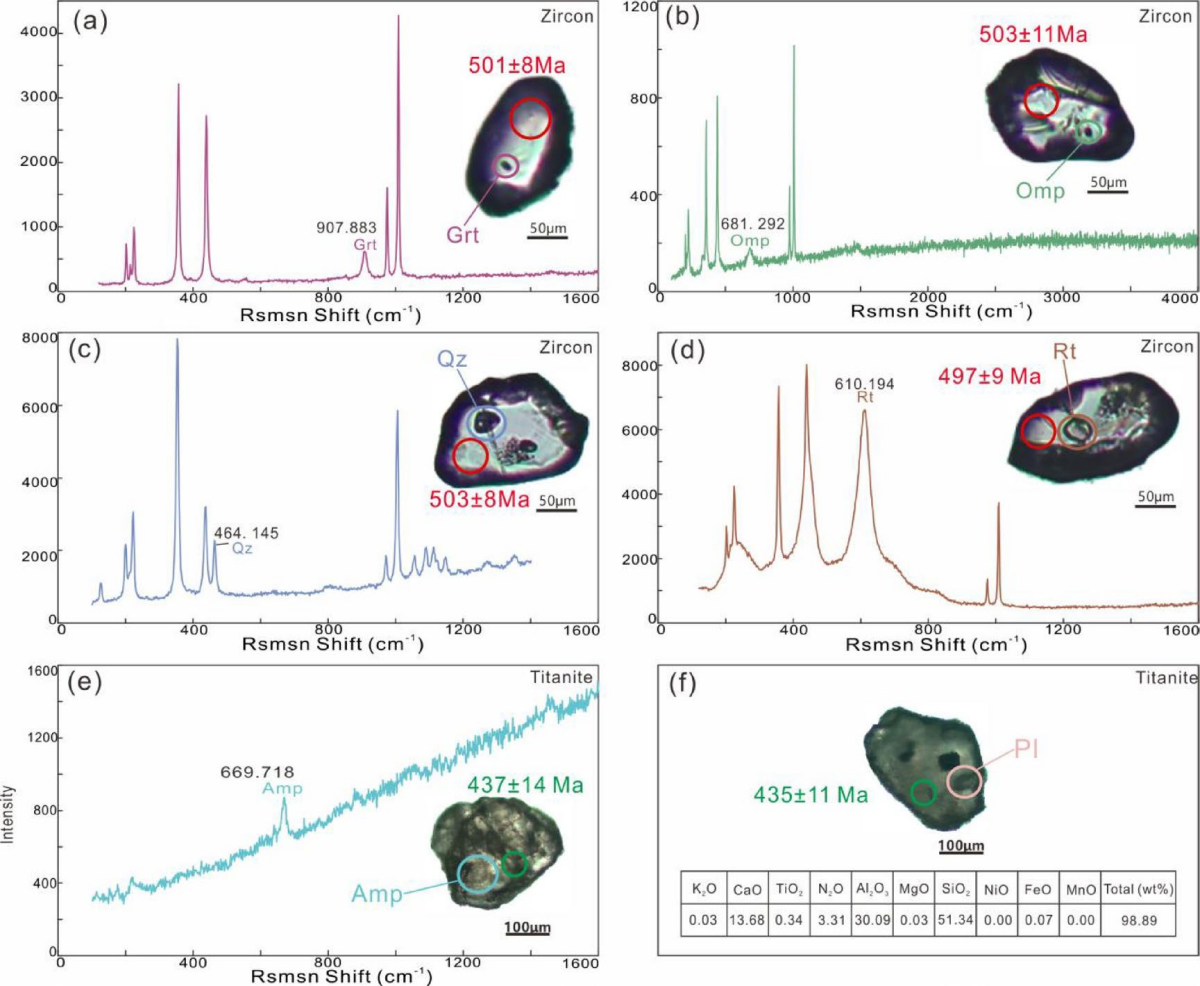
Representative mineral inclusions of Raman spectrum in zircons and titanites of the amphibolite from Munabulake (**a***–***e**) and EPMA major components of plagioclase inclusion in titanite (**f**). Note: the inclusions in b exhibit a Raman peak at 681 cm⁻¹ and lack an OH⁻-related peak around 3600 cm⁻¹, confirming their identity as omphacite rather than amphibole.

Inclusions in zircons and titanite were analysed by laser Raman spectroscopy. Garnet with a standard peak of 907.9 cm⁻¹ was found in the 501 Ma zircon domain (Fig. 9a); omphacite at 681.3 cm⁻¹ (Fig. 9b) and quartz at 464.1 cm⁻¹ (Fig. 9c) were found in 503 Ma domain; and rutile at 610 cm⁻¹ was found in 499 Ma domain (Fig. 9d). Amphibole with a standard peak of 669.7 cm⁻¹ was identified in the 437 Ma titanite domain (Fig. 9e); and plagioclase, confirmed by electron probe micro analysis (EPMA), was enclosed in 435 Ma domain (Fig. 9f).

## Phase equilibrium modelling

The calculated *P–T* pseudosection for amphibolite with effective bulk compositions for modelling are listed in Table S7, and were calculated using GeoPS software v3.6.3^52^ in the model system NCKFMASHTO. This approach utilizes the internally consistent thermodynamic dataset (updated version HP62) of Holland and Powell^53^ and accounts for the majority of the minerals present in the amphibolite. The a-x models for the main phases are as follows: garnet^54^, amphibole^55^, plagioclase^56^, ilmenite^57^ and silicate melt^55^. Rutile and quartz are regarded as pure end-member phases.

**Fig. 10:**
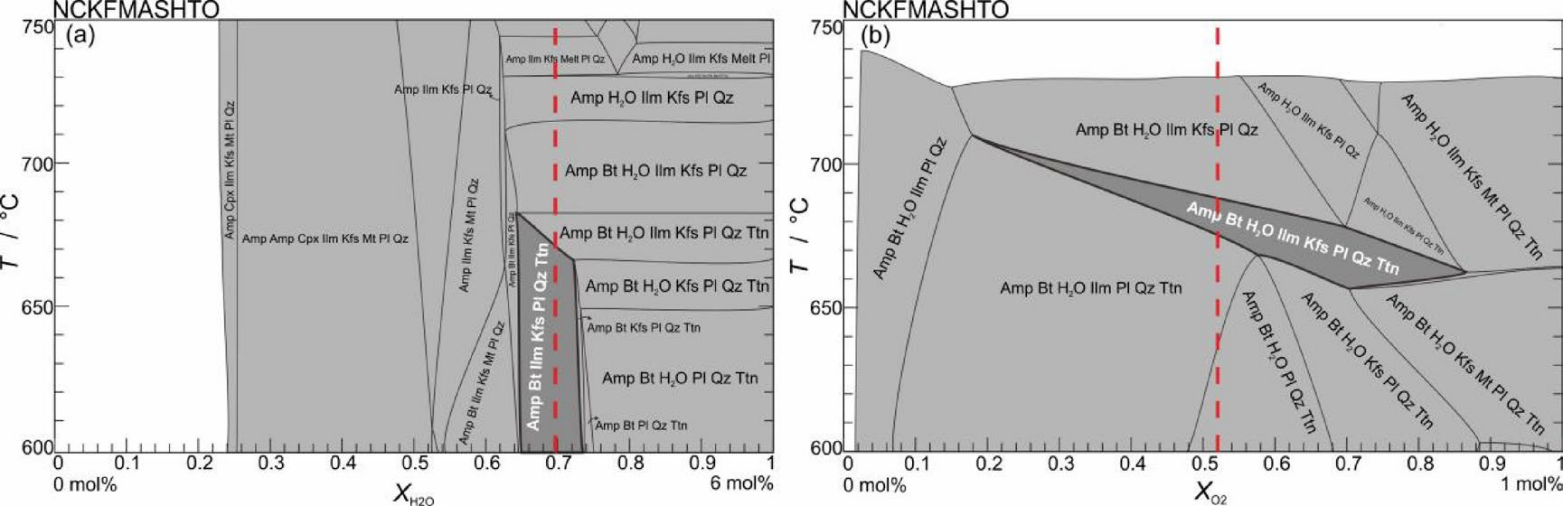
*T–X*_H2O_ and *T–X*_O2_ pseudosection of the amphibolite from Munabulake.

**Fig. 11:**
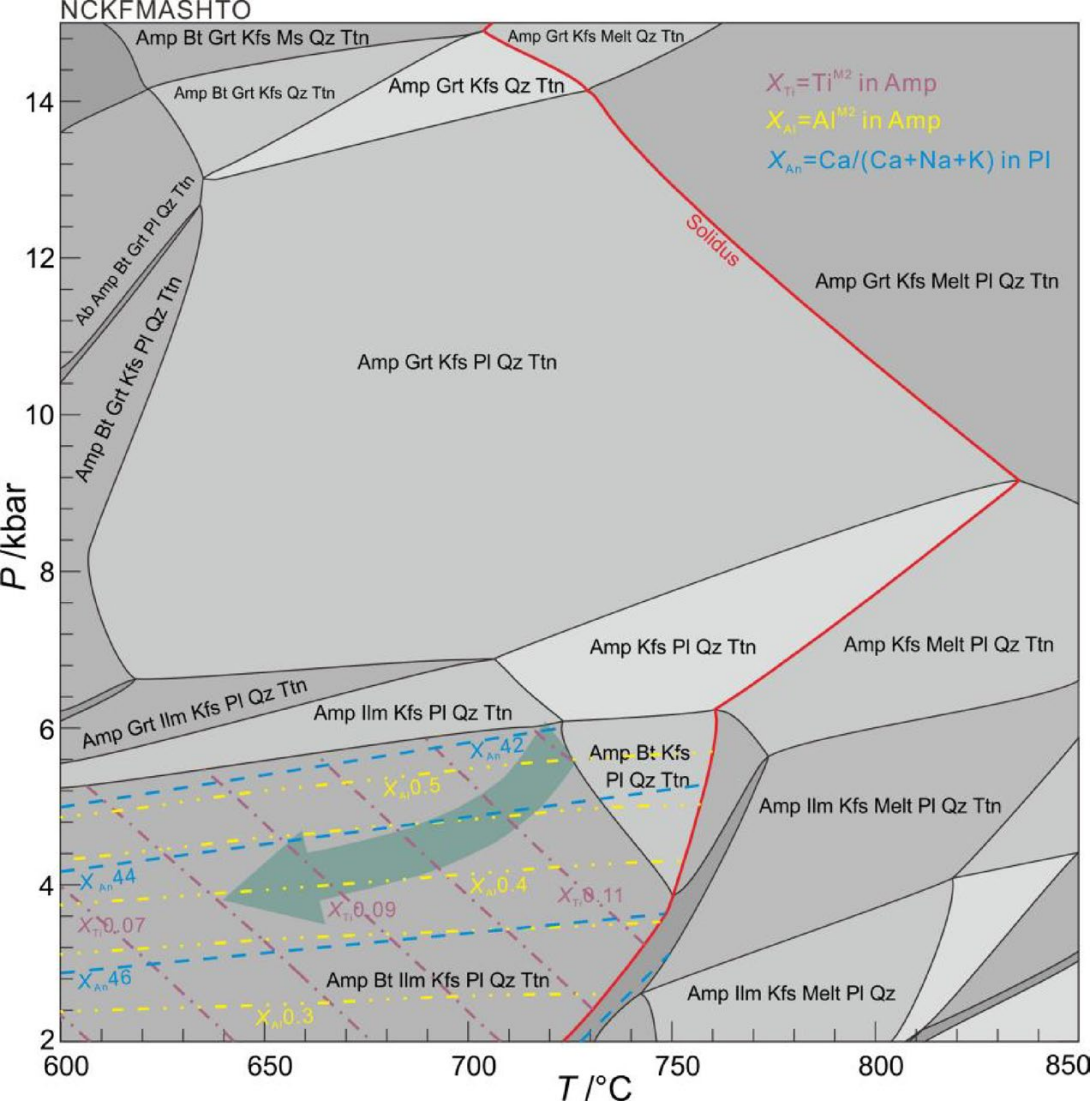
*P–T* pseudosection of the amphibolite from Munabulake.

Phase equilibrium modelling was conducted on the amphibolite using coupled *T–X*_H2O_ and *P–T* pseudosections. The *T–X*_H2O_ diagram was constructed with water content (H_2_O) ranging from 0 to 6 mol% (equivalent to *M*_H2O_ = 0–1; Fig. 10a). And the *T–X*_O_ diagram was constructed with oxygen content (O_2_) ranging from 0 to 1 mol% (equivalent to *X*_O2_ = 0–1; Fig. 10b). The correspondence observed minerals (Amp + Bt+Ilm + Kfs + Pl + Qz+Ttn) constrain the optimal *X*_H2O_ range to 0.65–0.74 and *X*_O2_ range to 0.17–0.88, from which the median *X*_H2O_ value of ~ 0.70 (corresponding to 4.2 mol% H_2_O) and *X*_O2_ value of ~ 0.52 (corresponding to 0.52 mol% O_2_) were adopted for computing the *P–T* equilibrium phase diagram.

In the *P*–*T* pseudosection (Fig. 11), the amphibolite assemblage (Amp + Bt + Ilm + Kfs + Pl + Qz + Ttn) is stable at low pressures (< 7.0 kbar) and temperatures (< 750 °C). Compositional variations in amphibole (Ti^M2^, Al^M2^) and plagioclase (An) define a more specific *P–T* range. The isopleths of *X*_Ti_ (X_Ti_ = Ti^M2^, 0.081–0.124) and *X*_Al_ (X_Al_ = Al^M2^, 0.45–0.58) yield *P* = 3.7–6.1 kbar and *T* = 640–725 °C; and the variational *X*_An_ (49–45) isopleths yield nearly identical results.

## Discussion

### Metamorphic *P*–*T*–*t* evolution

Zircon serves as a container of HP–UHP records due to its exceptional physicochemical resilience^58,59^. Zircons in HP–UHP terranes globally preserve key inclusion assemblages that fingerprint deep subduction processes. Coesite-bearing zircons in Dabie–Sulu and North Qinling document UHP metamorphism in retrogressed rocks^60,61^. North Qinling amphibolites yield zircons with Grt + Cpx + Qz inclusions^62^, while Polar Urals felsic gneisses’ zircons contain Na-Cpx + Grt inclusions^63^, collectively constraining eclogite-facies conditions. These findings highlight zircon’s unparalleled capacity to record peak metamorphic signatures erased in host rocks.

This study reconstructs a two-stage metamorphic *P–T–t* path for the amphibolite. (1) *P*-peak eclogite-facies stage. Eclogite-facies metamorphism is evidenced by integrated analyses despite the absence of preserved HP mineral assemblages in petrographic observations. Critical evidence includes: (i) diagnostic eclogite-facies mineral inclusions (Grt + Omp + Rt + Qz) within zircons, and (ii) metamorphic zircon signatures with low Th/U ratios (< 0.03) and flat HREE patterns lacking Eu anomalies, indicating crystallization under plagioclase-absent, garnet-stable eclogite-facies conditions^64,65^. The zircon age of 501.6 ± 2.7 Ma, constrained by these trace element and inclusion characteristics, reliably records the eclogite-facies metamorphic event. (2) Amphibolite-facies stage. The amphibolite-facies retrogression is evidenced by: (i) petrographic observations of the mineral assemblage Amp + Pl + Qz + Ilm, and (ii) titanite-hosted inclusions of Amp + Pl. Phase equilibrium modelling, constrained by amphibole (*X*_Ti_ = Ti^M2^/2; *X*_Al_ = Al – (8 – Si)) and plagioclase (*X*_An_ = An/(An + Ab+Or)), yields *P–T* conditions of 3.7–6.1 kbar and 640–725 °C for this stage. Decreasing Ti content from core to rim in amphiboles suggests near-isobaric cooling during retrogression. The titanite lower intercept age of 437 ± 6.2 Ma robustly dates the amphibolite-facies overprinting event.

**Fig. 12:**
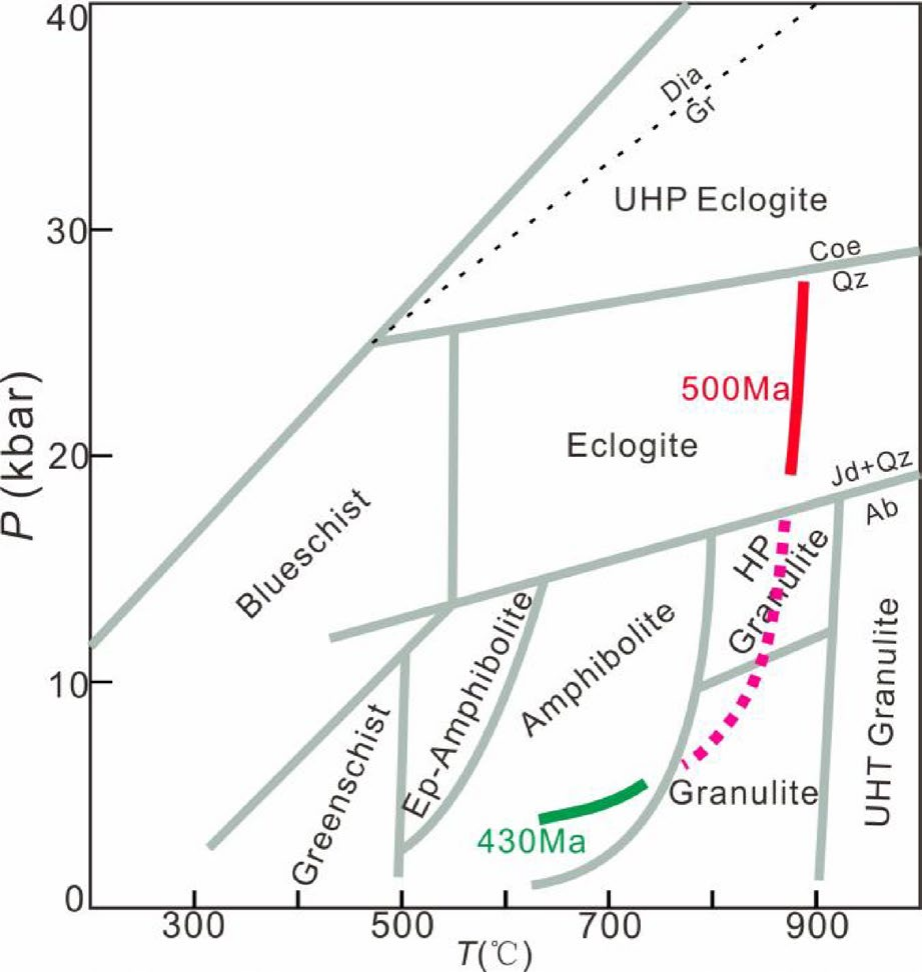
Integrated *P–T–t* path for the amphibolite from Munabulake.

In summary, the amphibolite records an initial eclogite-facies metamorphism at ~ 502 Ma, followed by amphibolite-facies overprinting at ~ 429 Ma, defining a clockwise *P–T–t* path with early near-isothermal decompression and late near-isobaric cooling (Fig. 12).

The Munabulake area, located in the westernmost SA HP–UHP metamorphic belt, was previously known for its HP granulites (*P*-peak; *P*>11Kbar, *T*>850℃; 485 Ma) and mica schist (498 ± 4 Ma)^29,44^. However, unlike other SA HP–UHP rocks, these rocks only recorded HP granulite-facies metamorphism. This study first identifies eclogite-facies metamorphism in local amphibolites, with U–Pb ages documenting *P*-peak (501.6 ± 2.7 Ma) and retrograde (437 ± 6.2 Ma) stages matching regional eclogite records^31^, suggesting shared subduction history despite extensive retrogression^66,67^.

### Protolith characteristics

Due to their high stability and insolubility, HFSEs, REEs, and transition elements generally remain unaffected during metamorphism, making them reliable tracers of protolith characteristics and tectonic setting of metamorphic rocks^68–70^.

**Fig. 13:**
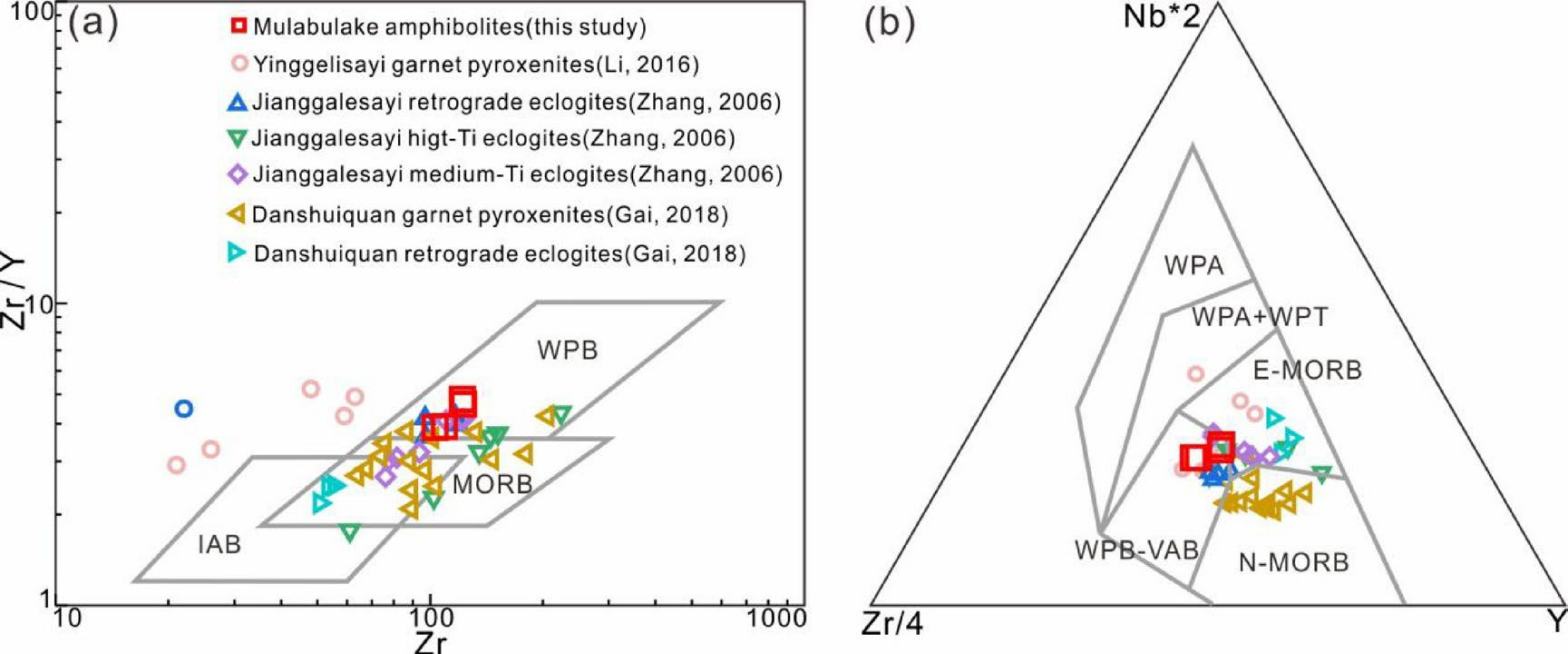
Tectonic discrimination diagrams of amphibolites, (**a**) Zr/Y versus Zr^74^, (**b**) Nb*2-Zr/4-Y^75^.

Previous studies classify the protoliths of SA metabasites into MORB-like and WPB-like. The medium-Ti eclogite from Jianggalesayi represents MORB-type^71^, showing Nb–Ta enrichment, Zr/Nb ratios of 9.9–13.7 (Zr/Nb > 16 for N-MORB and < 16 for E-MORB^72^, and La/Nb (average 0.82) and Y/Nb (average 3.2) ratios matching E-MORB values (MORB typically has La/Nb ≈ 0.76 and Y/Nb ≈ 3.5^73^), with all data plotting in E-MORB/MORB fields in Zr–Zr/Y and Nb–Zr–Y tectonic discrimination diagrams (Fig. 13). In contrast, retrograde eclogites represent WPB-type, exhibiting LREE-enriched patterns, Ta-U enrichment without Zr-Hf-Ti depletion, and consistent WPB-field positions in discrimination diagrams (Fig. 13).

The studied amphibolite exhibits subalkaline tholeiitic basalt characteristic, showing clear LILE enrichment (Rb, Th, U) without significant Nb–Ta anomalies, and LREE enrichment with flat HREE patterns (Figs. 5 and 6). Key diagnostic ratios (Ta/Hf = 0.19–0.23, Nb/Zr = 0.07–0.09, Th/Ta = 3.15–3.54, La/Nb = 1.03–1.15) and WPB-field positions in Zr–Zr/Y and Nb–Zr–Y diagrams (Fig. 13) consistently indicate a WPB affinity. These features suggest the protolith formed in an intraplate setting, potentially associated with Rodinia breakup during Neoproterozoic.

### Petrogenetic types of SA LP-MP rocks

Combining our results with previous studies, SA LP–MP rocks can be systematically classified into two genetic types:

#### Non–subduction-type

Greenschist-facies supracrustal rocks in SA (quartzite, mica/quartz/feldspar schist, and carbonate rocks) exhibit quartz + feldspar + biotite/muscovite assemblages, with garnet only in quartzite, indicating low-grade metamorphism^76^. Zircon U–Pb dating yields protolith ages of 1084–950 Ma, correlating with the Central Altyn Taxidaban Group^77^. These age evidence suggest that the greenschist-facies supracrustal rocks and the Taxidaban Group share a common crustal source and represent upper plate sedimentary units, supporting their classification as non-subduction products.

#### Retrograde modification-type

Microstructural and phase equilibrium studies reveal widespread HP–UHP records in SA LP–MP rocks, including: (a) Yaganbuyang garnet amphibolite shows omphacite-derived Cpx + Pl symplectites and garnet zoning, recording peak UHP conditions^28^; (b) Jianggalesayi pelitic gneiss with quartz-hosted spinel + kyanite rods suggests former stishovite (~ 300 km depth)^21^; (c) Bashiwake granitic gneiss exhibits rod-shaped plagioclase + amphibole exsolution textures in titanite, suggesting peak metamorphic pressures of 3.7–4.3 GPa^78^. Additionally, our study identified garnet + clinopyroxene inclusions in amphibolite’s zircons and zircon flat HREE patterns indicative of eclogite-facies conditions.

Collectively, these rocks underwent HP–UHP metamorphism followed by retrogressive overprinting during exhumation, accounting for their current LP–MP characteristics and supporting retrograde overprint model.

### Geological significance

**Fig. 14:**
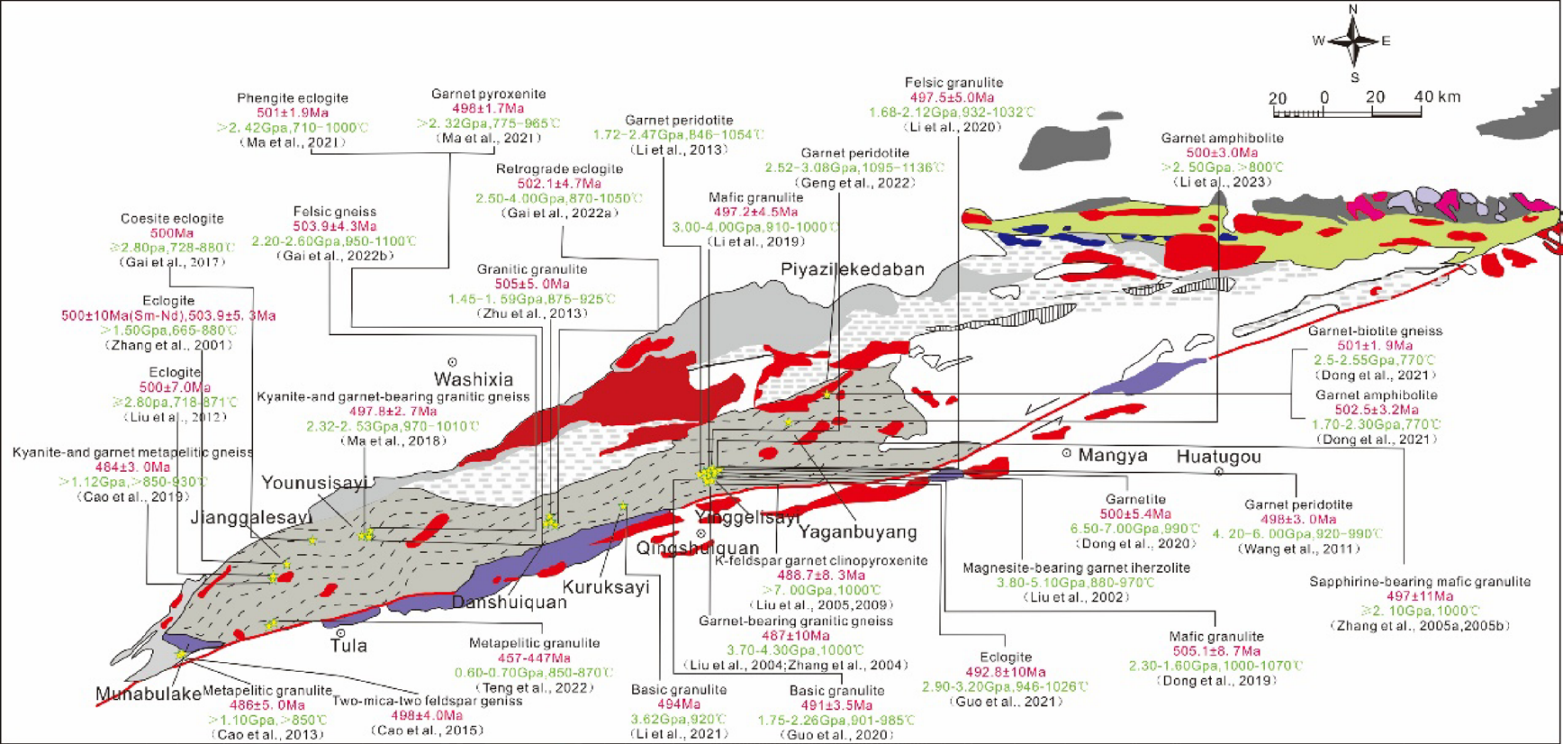
Summary diagram of peak metamorphic conditions and ages of metamorphic rocks in the South Altyn Tagh (geological map modified after Wang et al.^34^).

Since the eclogite discovered in Jianggalesayi^79^, the SA has rapidly emerged as a critical locality for studying continental deep–ultradeep subduction and HP–UHP metamorphism. Subsequent studies have identified extensive HP–UHP exposures (eclogites, granulites, gneisses, garnet clinopyroxenites/peridotites) spanning ~ 350 km from Munabulake to Piyazikele (Fig. 14)^20,23,25–29,31,40,41,43,44,78,80–95^. These rocks öor exceeding the minimum eclogite-facies conditions^96^ and consistently yield peak/near-peak metamorphic ages of ~ 500 Ma, indicating that the SA likely experienced unified Early Paleozoic continental deep–ultradeep subduction.

While these rocks record comparable peak metamorphic ages, they exhibit significant variations in pressure conditions, mineral assemblages, and preservation states. Such discrepancies likely reflect heterogeneities in both the exhumation processes and the intensity of retrograde overprinting^16,97^. For instance, the eastern SA records HP–UHT retrograde metamorphism (~ 2.0 GPa/870–1050 °C) with a short ~ 16 Myr interval between UHP eclogite-(c. 500 Ma) and granulite-facies (c. 484 Ma) metamorphism, contrasting with the western SA’s near-isothermal UHP decompression and cooling (no UHT overprinting) with a longer ~ 45 Myr eclogite- (c. 500 Ma) to HP granulite-facies (c. 455 Ma) gap, revealing differential exhumation processes between the eastern and western SA segments^26^.

In addition, the retrograde overprinting intensity in SA HP–UHP rocks shows regional variability, primarily controlled by: (1) Structural deformation^98^– weakly deformed felsic gneisses preserve full *P–T* paths (peak, 2.2–2.6 GPa, 950–1100 °C), while strongly deformed ones only retain retrograde assemblages (0.87–1.1 GPa, 750–770 °C), suggesting that deformation plays a dominant role during retrogression^99^; (2) Partial melting^98,100^– leucosome-bearing gneisses experienced enhanced retrogression due to melt-weakened rheology during rapid exhumation, which also facilitating rapid exhumation^101^. The specific controlling factors require further investigation in future studies.

## Conclusions

1. This study reports the first occurrence of eclogite-facies metamorphism in the westernmost SA Munabulake area, dated at 501.6 ± 2.7 Ma and identified by garnet + omphacite inclusions and specific REE patterns in zircon. Titanite-hosted amphibole + plagioclase inclusions record amphibolite-facies retrogression at 437 ± 6.2 Ma, with estimated *P–T* conditions of 3.7–6.1 kbar and *T* = 640–725 °C.
2. The amphibolite’s protolith exhibits calc-alkaline tholeiitic basalt affinities, consistent with the widely distributed retrograde eclogites in the region.
3. The current mixed spatial-temporal distribution of variably-graded metamorphic rocks in the SA results from diverse exhumation mechanisms and varying degrees of retrograde metamorphic overprinting.

## Supplementary Information

Below is the link to the electronic supplementary material.

Supplementary Material 1

Supplementary Material 2

## Data Availability

All data generated or analysed during this study are included in this published article [and its supplementary information files].
