# Supplementary material for: Metamorphic evolution of amphibolite from Proto-Tethys South Altyn orogen and its geological significance
Source: Sci Rep. 2026 Mar 17;16:13819. doi: 10.1038/s41598-026-44259-0 (PMC13129027; doi:10.1038/s41598-026-44259-0)
Supplement: Supplementary file 2 — Supplementary Material 2 [file 41598_2026_44259_MOESM2_ESM.doc]

**Metamorphic evolution of amphibolite from Proto-Tethys South Altyn orogen and its geological significance**

**Shihao Zhang ^a^, Tuo Ma ^a*^, Yongsheng Gai** **^a^, Liang Liu ^a^**

^a^ State Key Laboratory of Continental Evolution and Early Life, Department of Geology, Northwest University, Xi’an 710069, China

* Corresponding authors

E-mail address: matuo12@163.com (Tuo Ma)

**S1** **Experimental methods**

**S1.1 Mineral** **compositions**

Quantitative mineral compositions were determined using a JEOL JXA-8230 electron probe microanalyser (EPMA) at Northwest University. Analytical conditions included an accelerating voltage of 15 kV, a probe current of 10 nA, and a beam diameter of 1 μm. Calibration was performed using SPI standards: jadeite (Si, Al, Na), ilmenite (Fe), rhodonite (Mn), rutile (Ti), olivine/pyrope (Mg), diopside (Ca), and Sanidine (K).

**S1.2 Bulk-rock compositions**

Whole-rock major element compositions were measured using a Rigaku RIX 2100 X-ray fluorescence (XRF) spectrometer. Trace element concentrations were determined by inductively coupled plasma mass spectrometry (ICP-MS). Analytical accuracy and precision were monitored through the USCG standards BHVO-1, AVG-1, and BCR-2.

**S1.3 Zircon U-Pb dating and trace-element**

Zircon grains were separated using conventional heavy liquid and magnetic separation. Cathodoluminescence (CL) imaging was conducted using a Quanta 400FEG environmental scanning electron microscope equipped with an Oxford energy-dispersive spectroscopy system and a Gatan CL^3+^ detector. Zircon trace element concentrations and U–Pb isotopic ratios were analysed using LA-ICP-MS system. Both trace elements and U–Pb isotopes were acquired simultaneously from the same ablation spots. Analytical conditions included a spot diameter of 32 μm, an ablation depth of ~20 μm, and He as the carrier gas. The international zircon standard 91500 was used as the external standard for U–Pb dating with a recommended ^206^Pb/^238^U age of 1065.4±0.6 Ma^1^. The synthetic silicate glass NIST SRM 610 was used for instrument calibration, and ^29^Si was employed as an internal standard for quantifying elemental concentrations. Data reduction for U–Pb isotopic ratios and trace elements was performed using the ICPMSDataCal 10.8 software^2,3^, while concordia diagrams and weighted mean age calculations were carried out using Isoplot 3.23^4^.

**S1.4 Titanite U-Pb dating and trace-element**

Titanite U–Pb isotopic analyses were conducted using LA-ICP-MS at Wuhan Sample Solution Analytical Technology company, People’s Republic of China. Trace element calibration was performed using the NIST SRM 610 glass standard, while isotopic ratios were calibrated against the zircon standard 91500 and monitored using the MKED1 reference material. Analytical conditions included a laser energy of 80 mJ, a repetition rate of 3 Hz, and a spot size of 44 μm. Detailed analytical procedures are described in Luo et al.^5,6^. Data reduction for titanite U–Pb isotopic ratios was conducted using the same software as that used for zircon analyses.

**S1.5 Inclusion analyses**

Inclusion analyses in zircon and titanite were performed using a Renishaw Invia laser Raman spectrometer (Renishaw, High-Power NIR Laser, UK) with a 514.5 nm Ar⁺ laser as the excitation source. The laser beam diameter was 1–2 μm, and the laser power on the sample surface was maintained at approximately 30 mW. A 50× objective lens on a Leica DM LM microscope was used, and a grating with 1800 grooves per mm was selected to achieve high spectral resolution. Each spectrum was collected over a 0–1600 cm⁻¹ range, with an exposure time of 10 s and three accumulations (total = 60 s) per analysis spot.

To ensure data consistency, instrument drift was routinely monitored by repeated measurements of the 520 cm⁻¹ band of silicon and the 1008 cm⁻¹ band of undoped synthetic zircon. The obtained Raman spectra were fitted using Wire 2.0 software with a Lorentz–Gaussian function to determine the Raman shift and full width at half maximum (FWHM) parameters. Detailed analytical methods are described in Fan et al.^7^.

**Reference**

1. Wiedenbeck, M., Hanchar, J. M., Peck, W. H. *et al.* Further characterisation of the 91500 zircon crystal. *Geostand. Geoanal. Res.* **28**, 9–40 (2004).
2. Liu, Y., Gao, S., Hu, Z. *et al.* Continental and oceanic crust recycling-induced melt–peridotite interactions in the Trans-North China Orogen: U–Pb dating, Hf isotopes and trace elements in zircons from mantle xenoliths. *J. Petrol.* **51**, 537–571 (2010).
3. Liu, Y., Hu, Z., Gao, S. *et al.* In situ analysis of major and trace elements of anhydrous minerals by LA-ICP-MS without applying an internal standard. *Chem. Geol.* **257**, 34–43 (2008).
4. Ludwig, K. R. User’s manual for IsoPlot 3.0: A geochronological toolkit for Microsoft Excel. *Spec. Publ., Berkeley Geochronol. Center* **71**, 1–70 (2003).
5. Luo, T., Hu, Z., Zhang, W. *et al.* Reassessment of the influence of carrier gases He and Ar on signal intensities in 193 nm excimer LA-ICP-MS analysis. *J. Anal. At. Spectrom.* **33**, 1655–1663 (2018).
6. Luo, T., Hu, Z., Zhang, W. *et al.* Water vapor-assisted “universal” nonmatrix-matched analytical method for the in situ U–Pb dating of zircon, monazite, titanite, and xenotime by laser ablation-inductively coupled plasma mass spectrometry. *Anal. Chem.* **90**, 9016–9024 (2018).
7. Fan, M., Liu, X., Sun, S. et al. Effect of chemical composition on zircon radiation damage dating: Implications for low-temperature thermochronology. *Geosci. Front.* **14**, 101675 (2023).
